# Omega-3 Fatty Acids for Sport Performance—Are They Equally Beneficial for Athletes and Amateurs? A Narrative Review

**DOI:** 10.3390/nu12123712

**Published:** 2020-11-30

**Authors:** Frank Thielecke, Andrew Blannin

**Affiliations:** 1 Department of Health, Swiss Distance University of Applied Sciences, 8105 Regensdorf-Zürich, Switzerland; 2 Thielecke Consultancy, Bettenstrasse 60a, 4123 Allschwil, Switzerland; 3 Exercise and Rehabilitation Sciences, School of Sport, University of Birmingham, Birmingham B15 2TT, UK; a.k.blannin@bham.ac.uk

**Keywords:** omega-3 fatty acids, sports nutrition, athletes, amateurs, performance, recovery, injury

## Abstract

Omega-3 fatty acids, specifically eicosapentanoic acid (EPA, 20:5n-3) and docosahexanoic acid (DHA, 22:6n-3) are receiving increasing attention in sports nutrition. While the usual focus is that of athletes, questions remain if the different training status between athletes and amateurs influences the response to EPA/DHA, and as to whether amateurs would benefit from EPA/DHA supplementation. We critically examine the efficacy of EPA/DHA on performance, recovery and injury/reduced risk of illness in athletes as well as amateurs. Relevant studies conducted in amateurs will not only broaden the body of evidence but shed more light on the effects of EPA/DHA in professionally trained vs. amateur populations. Overall, studies of EPA/DHA supplementation in sport performance are few and research designs rather diverse. Several studies suggest a potentially beneficial effect of EPA/DHA on performance by improved endurance capacity and delayed onset of muscle soreness, as well as on markers related to enhanced recovery and immune modulation. The majority of these studies are conducted in amateurs. While the evidence seems to broadly support beneficial effects of EPA/DHA supplementation for athletes and more so in amateurs, strong conclusions and clear recommendations about the use of EPA/DHA supplementation are currently hampered by inconsistent translation into clinical endpoints.

## 1. Introduction

The main purpose of nutrition for athletes is to compensate for increased energy and nutrient needs. In recent years, the role of omega-3 fatty acids in sport has received increasing research attention [1]. Omega-3 fatty acids are perceived as a potential supplement that may beneficially affect performance, recovery and the risk for illness/injury [2]. Omega-3 fatty acids belong to the family of polyunsaturated fatty acids [3]. While there are fatty acids of varying length, the most important ones are considered to be the very long-chain fatty acids eicosapentanoic acid (EPA, 20:5n-3) and docosahexanoic acid (DHA, 22:6n-3) [4]. The predominant source for EPA/DHA is seafood, particularly fatty fish, such as mackerel and herring. Although food items such as linseed oil and walnut oil have high amounts of the plant-derived omega-3 fatty acid α-linolenic acid (ALA, 18:3n-3) they are not routinely consumed in large quantities. Other food products, such as soybeans, squash and wheat germ cereals contain less ALA but are often consumed in higher amounts and therefore contribute significantly to ALA intake. While EPA can be synthesized from ALA, the conversion of ALA to EPA and further to DHA is characterized by a low conversion rate [5], therefore the consumption of EPA/DHA via seafood is generally recommended. Although the current recommendations stand, it should be noted that there is a substantial genetic variation in the fatty acid metabolism [6].

In the European Union (EU), across all populations, EPA and DHA intake is not adequate; in 74% of the EU countries the intake was found to be lower than the European Food Safety Agency (EFSA) recommendation of 250 mg for EPA and DHA as adequate intake for adults based on cardiovascular considerations [7,8]. The current dietary guidelines for Americans suggest the same value [9]. EPA/DHA are considered safe up to 5 g per day [10]. Although EFSA’s recommendation is for the normal, healthy population, it is reasonable to assume that neither athletes nor amateurs consume adequate amounts either considering their higher energy turnover and metabolic flux. In fact, analyses of dietary habits in various athletes found that substantial proportions of the studied populations did not reach the dietary goals for macro- and micronutrients, including EPA/DHA [11,12]. Furthermore, a recent multi-center, cross-sectional study in 404 National Collegiate Athletic Association Division I football athletes revealed that no athlete had an Omega-3 Index associated with low risk [13].

While there are some data that EPA/DHA may improve endurance capacity and promote recovery in athletic populations [14], current evidence lacks consensus [1]. Given the fact that a lot of studies with omega-3 fatty acids were conducted in non-professionals, we also include studies conducted in amateurs (defined as people pursuing an activity for pleasure without payment and not as a job). Broadening the data base may shed more light on the effects of EPA/DHA supplementation on performance parameters. Furthermore, this large section of the population is also of interest as it can potentially benefit from an optimized diet as well. Studies were included when parameters relevant for performance, recovery and risk of illness/injury were reported. Hence, this narrative review identifies relevant human intervention studies and evaluates the overall impact of EPA/DHA in sport nutrition for athletes as well as amateurs. The scope of this review is to provide a simplified and exploratory, yet relevant approach to assess the role of EPA/DHA on outcomes related to performance, recovery and illness/injury in two different populations, athletic as well as amateurs, by using dichotomous splits to describe the study outcomes focusing on study duration and dose. The differentiation between athletes and amateurs is important, because the metabolic state, i.e., training status may well influence the response to a given stimulus or supplementation. In general, varying designs particularly in the dose of EPA/DHA as well as the duration of supplementation contribute substantially to the partly inconsistent outcomes. Our approach enabled us to identify differences in outcomes related to dose and duration between athletes and amateurs, which may translate not only into tailored intake recommendations but into design considerations for future clinical trials to assess the efficacy of EPA/DHA supplementation in performance.

## 2. Materials and Methods

We conducted a narrative literature review and search using the PubMed database with predefined keywords, as well as MeSH terms. The literature search was finalized in March 2020. The search strategy included the terms: (omega-3 fatty acids OR n-3 fatty acids OR fish oil) AND (sport OR sports OR performance). The selection criteria were randomized controlled clinical trials that were published between January 2010 and February 2020. The search was limited to humans, and the English language. Initially, 310 articles were retrieved. After screening of titles and abstracts, 52 papers were selected for further examination. In addition, 1 article was identified by subsequent hand search, so that 53 articles reporting on randomized controlled trials over the last decade in both athletes as well as amateurs were included in this narrative review. We wanted to explore whether the dose or duration of the supplementation was influencing the findings. However, these factors were not normally distributed across the studies so we decided to adopt a simple dichotomized approach to enable us to compare the highest against the lowest for both dose and duration [15].

## 3. Results

**Figure 1:**
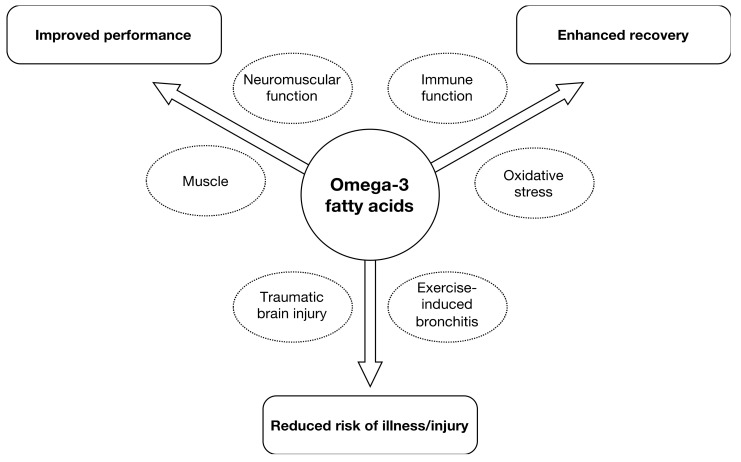
Areas of interest for supplementation with eicosapentanoic acid/docosahexanoic acid (EPA/DHA) in sport nutrition in athletes as well as amateurs.

EPA and DHA can affect many aspects of human physiology/metabolism and these can subsequently impact outcomes related to sporting performance, recovery and illness/injury is shown in Figure 1.

Of all the studies that were identified for this review, multiple outcomes were often reported within each study, which in some cases made allocation to only one of the main topics a challenge (performance, recovery or illness/injury). Hence the allocation of studies to performance, recovery or illness was based on evaluation of the main outcome reported. Overall, of the 53 articles, 21 articles reported on athletes and 32 in amateurs.

### 3.1. The Influence of Dose and Study Duration in Athletes and Amateurs on Performance

Thirty studies were identified that assessed the effects of EPA/DHA supplementation with a focus on performance-related outcomes in athletes and amateurs (Table 1). One study was used twice because of 2 sets of data (low dose and high dose groups). Ten of those studies were conducted in athletes, with 21 in amateurs. The amounts used in the studies reviewed here vary for EPA from 0.06 g to 4.9 g per day and for DHA from 0.04 to 4.7 g DHA per day. The duration of supplementation ranged from acute to 24 weeks.

A recent study in 26 competitive soccer players supplemented with 4.9 g EPA and 1.4 g DHA per day over 4 weeks found that the increases in leg strength, sprint speed, explosive power and anaerobic endurance were not different between groups [20]. No benefits of supplementation with 0.56 g EPA and 0.14 g DHA over 8 weeks were found on power output and MVC in a group of trained males [23]. In contrast, explosive power, fatigue and muscle soreness were improved in athletes consuming 1.1 g of each EPA and DHA over 5 weeks [23]. Similarly, in a study with trained males, squat jump performance was improved after a single acute supplementation with 0.75 g EPA and 0.05 g DHA [24]. A greater number of studies have been conducted in amateurs. Muscle strength was increased in long-term supplementation studies with 0.4 g EPA and 0.3 g DHA over 21 weeks, or 1.86 g EPA and 1.5 g DHA over 24 weeks [26,27]. Mixed results were reported from studies in amateur males with 0.375 g EPA and 0.51 g DHA [41,42] where the latter reported no benefits on strength but improved fatigue. A short-term supplementation with 2 g EPA and 1 g DHA over one week did not demonstrate any effects on arm circumference or volume [46]. No improvement in strength was found in amateur males and females after 4 weeks of 2.7 g fish oil [39]. Muscle protein synthesis, was unchanged after 3.5 g EPA and 0.9 g DHA over 8 weeks [31].

**Figure 2:**
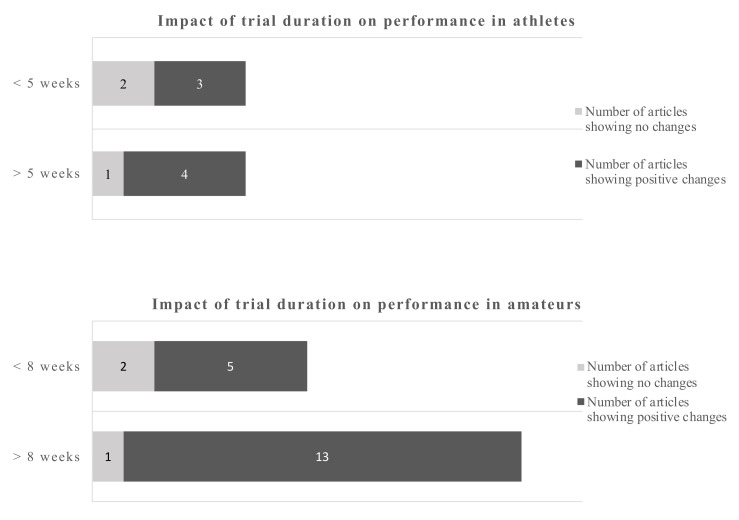
Impact of trial duration on performance-related outcomes in athletes and amateurs by dichotomous split.

Anaerobic endurance improved after supplementation with 4.9 g EPA and 1.4 g DHA over 4 weeks in a group of soccer players [20]. Others reported beneficial results in male athletes in various parameters relevant for endurance such as submaximal exercise HR and O_2_ consumption VO_2max_ and relative O_2_ consumption [16,23] (Table 1). Others found diastolic blood pressure and HR during submaximal exercise decreased in athletes, supplemented with 1.9 g EPA/DHA, but these changes did not translate in delayed time to exhaustion during a run, nor to enhanced recovery [19]. While the above changes could result in enhanced performance, the time to voluntary fatigue was not different between groups in a comparable study [16]. Nor did athletes exercising during a 1-h time trial [22] or a 10-km time trial [17] show a beneficial effect of supplementation with EPA/DHA. In amateur males, no effects on cardiac output at rest or during an exercise stress test were found after acute supplementation with either 4.7 g EPA or 4.7 g DHA, but systemic vascular resistance was reduced following DHA supplementation only [45]. An earlier study found neither substrate oxidation, energy expenditure nor energy efficiency to be affected by 2-week supplementation with 1.1 g EPA and 0.7 g DHA in amateurs [43]. However, a study in amateurs in which EPA/DHA were administered in doses of 0.6 g and 0.3 over 8 weeks, led to a significant increased VO_2max_ [38]. An improved O_2_ uptake during submaximal exercise in amateurs after supplementation with 0.9 g EPA and 0.4 g DHA was confirmed by others [30,32]. The latter study was based on cardiovascular parameters in response to submaximal exercise with amateur overweight adults [30]. Similarly, amateurs who had received 0.8 g EPA and 2.4 g DHA over 8 weeks showed significantly lower heart rates during incremental work load up to exhaustion, lowered steady-state submaximal exercise heart rates and increased whole body O_2_ consumption [16]. Also smaller amounts (0.56 g DHA and 0.14 g EPA) tended to reduce mean exercise HR and improved HR recovery in amateurs [35]. The influence of study duration on performance-related outcomes is depicted in Figure 2.

Using a cut-off of 5 weeks for athletes and 8 weeks for amateurs gives a dichotomous split. The evidence favors longer trials which is more pronounced in amateurs.

**Figure 3:**
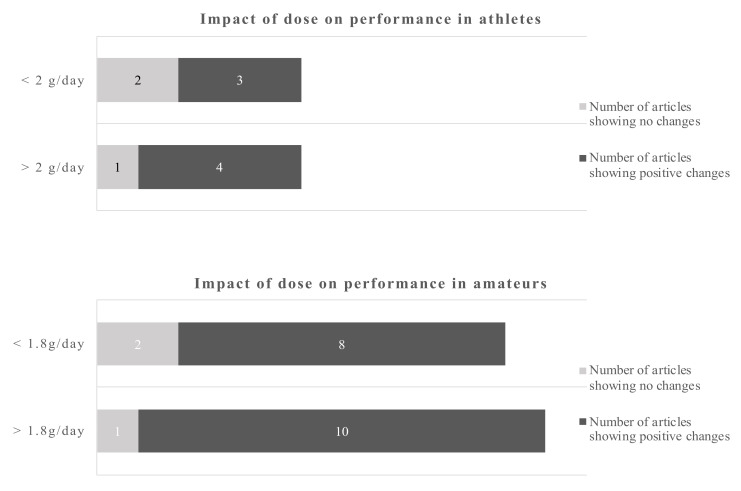
Impact of dose on recovery-related outcomes in athletes and amateurs by dichotomous split.

Using a dichotomous split for each, athletes and amateurs, the cut-off points were 5 weeks and 8 weeks respectively. For athletes, 4 out of 5 studies and 13 out of 14 studies in amateurs, favored the longer duration studies in terms of providing positive performance outcomes (Figure 3).

Using a cut-off of 2 d/day for athletes and 1.8 g/day for amateurs gives a dichotomous split. The evidence favors the higher doses which is more pronounced in amateurs. However, even the lower dose gives more positive changes as opposite to the low dose in athletes.

The data in amateurs were more pronounced. The cut-off points for the dose were 2 g/day for athletes and 1.8 g/day for amateurs. The data suggest that doses below 2 g/day in athletes sometimes induce a beneficial outcome, while above that cut-off point 4 out of 5 studies showed beneficial outcomes. The dose in amateurs appear to be of less influence, as 18 out of 21 studies reported a beneficial outcome regardless whether above or below the cut-off point.

### 3.2. The Influence of Dose and Study Duration in Athletes and Amateurs on Recovery

Twenty-two studies were identified that assessed the effects of EPA/DHA supplementation with a focus on recovery-related outcomes in athletes and amateurs Table 2. 

Eleven of those studies were conducted in athletes, 11 in amateurs. The amounts used in the studies reviewed here varied for EPA from 0.06 g to 2.4 g per day and for DHA from 0.04 to 1.2 g DHA per day. The duration of supplementation ranged from acute to 24 weeks (Table 2).

A recent study in 30 male athletes found that 6 weeks of supplementation with 0.55 g EPA and 0.55 g DHA reduced muscle soreness after eccentric exercise, without an effect on muscle function [54]. Reduced soreness in amateurs due to supplementation with EPA/DHA has been consistently reported [34,40,46]. Soreness following eccentric exercise was also reported less in amateurs who received 3 g of DHA for a little more than a week [63]. Furthermore, delayed onset of muscle soreness was also reported after 8 weeks of 0.6 g EPA and 0.26 g DHA [33], or 4 weeks of 2.7 g of fish oil [39]. An intervention study in 27 amateur males showed no effect of 1.8 g/d omega-3 fatty acids on knee ROM, perceived pain, and thigh circumference when measured immediately, and after 24 h of eccentric exercise [58]. However, perceived pain and ROM were improved at 48 h post-exercise. In contrast, participants who were able to achieve full elbow extension improved after 1 week of supplementation with 3 g DHA, while passive extension or arm swelling were not [63]. Others found a trend for reduced soreness after eccentric exercise in amateur females [44].

In young, but not older athletes, pro-inflammatory gene expression in response to exercise were increased following 5 weeks supplementation with 0.83 g DHA, in combination with alpha-tocopherol [55]. In contrast, decreased inflammatory responses following intense exercise were reported in a group of athletes that received doses of 1.2 g EPA and 2.4 g DHA [57]. Partially beneficial results were reported after 1.16 g DHA for 8 weeks by exerting anti-inflammatory effects via increasing plasma PGE_2_ [49]. Furthermore, exercise-induced increases in various cytokines, including interleukin 6 and 8, were decreased following supplementation with 1.16 g DHA for 8 weeks [50], while one study found interleukin 4 and 6 remained unaffected in amateurs that received 1.3 g EPA and 0.3 g DHA for 6 weeks [60].

**Figure 4:**
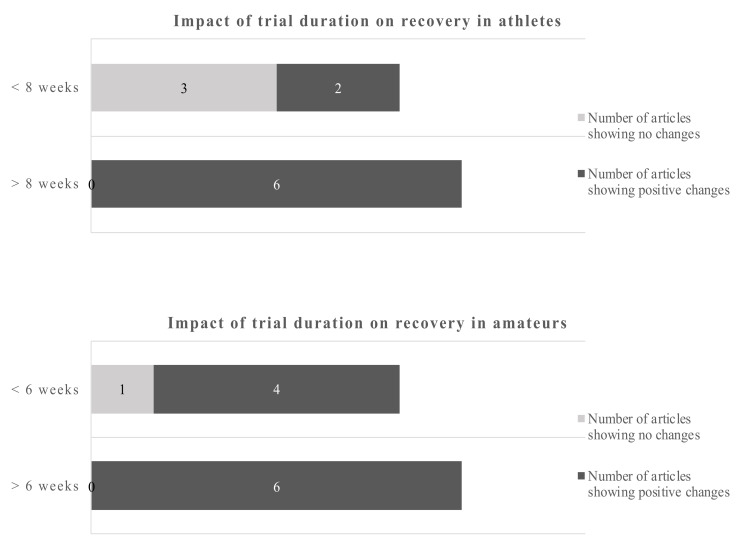
Impact of trial duration on recovery in athletes and amateurs by dichotomous split.

In athletes, improved antioxidant capabilities in response to acute exercise were reported after supplementation with 1.14 g of DHA over 8 weeks [48]. A slightly higher dose of DHA over a longer period of time decreased exercise-induced peroxidative damage [52]. However, using the same supplementation protocol, the authors showed increased markers of oxidative damage during training [51]. Potentially aggravating effects were also reported in athletes with increased oxidative stress at rest and after training following the consumption of 0.6 g EPA and 0.4 g DHA [65]. In a further study, 0.82 g of DHA in combination with 0.33 g alpha-tocopherol showed neither an effect on oxidative damage following a maximal exercise test nor changes in the antioxidant gene expression [55]. In amateurs, 1.3 g EPA and 0.3 g DHA reduced certain markers of oxidative stress after a single bout of exercise, while other parameters, including endogenous DNA damage and muscle soreness, were unaffected [61]. Regarding study duration the cut-off points were 8 weeks for athletes and 6 weeks for amateurs (Figure 4).

Using a cut-off of 8 weeks for athletes and 6 weeks for amateurs gives a dichotomous split. The evidence favors longer trials in athletes and amateurs.

**Figure 5:**
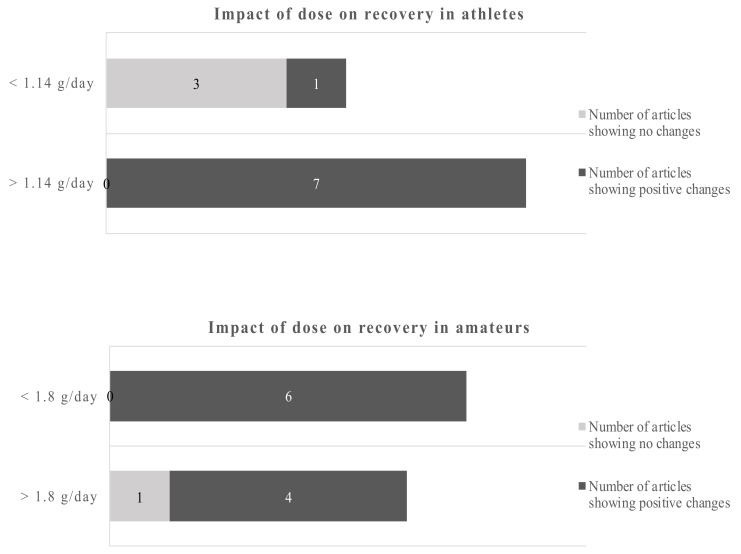
Impact of dose on recovery in athletes and amateurs by dichotomous split.

Studies of less than 8 weeks duration in athletes showed no clear picture of the benefits of omega-3 fatty acids, only 2 out of 5 studies reported beneficial outcomes. However, 6 out of 6 studies with more than 8 weeks reported positive changes due to the EPA/DHA supplementations. Studies in amateurs showed positive changes of the omega-3 fatty acids in 4 out of 5 studies below 6 weeks of duration and 6 out of 6 studies of more than 6 weeks duration. Applying the dichotomous approach on the dose, the observed cut-off points for athletes and amateurs were 1.14 g/day and 1.8 g/day, respectively. One out of 4 studies reported beneficial effects of the EPA/DHA supplementation in athletes when the supplementation was lower than 1.14 g/day. However, all studies with a dose above 1.14 g/day showed beneficial effects. In amateurs, 10 out of 11 studies showed beneficial effects of EPA/DHA supplementation on recovery-related outcomes (Figure 5).

Using a cut-off of 1.14 g/day for athletes and 1.8 g/day for amateurs gives a dichotomous split. Overall, the data are clearer for amateurs. The evidence favors higher doses in athletes and amateurs.

Overall, the evidence shows that EPA/DHA have the potential to decrease the production of inflammatory eicosanoids, cytokines, and ROS. Amateurs appear to benefit more; particularly soreness is beneficially affected by supplementation with EPA/DHA (Table 2).

### 3.3. Reduced Risk of Injury/Illness

Four studies were identified that assessed the effects of EPA/DHA on injury/illness (Table 3).

Two of those studies report on athletes and two on amateurs. A study in trained males showed similar improvements in markers of pulmonary function, albeit with a much higher dose of up to 3.7 g EPA and 2.5 g DHA over 3 weeks [67]. Improved markers for pulmonary function, including hyperpnea-induced bronchoconstriction, a surrogate for exercise-induced bronchoconstriction (EIB), were also observed after 3 weeks of a low daily dose of 0.07 g EPA and 0.05 g DHA during an eucapnic hyperventilation challenge in amateurs [68], with EPA/DHA possibly acting as an inflammatory antagonist. In contrast, no changes in inflammatory markers were found in a study in amateur males and females with a high dose of 4 g EPA and 2 g DHA over 3 weeks [69].

Evidence from observational and intervention studies suggests a beneficial effect of EPA/DHA in asthma in the general population [70,71,72]. An early intervention study in athletes showed that fish oil supplementation reduced exercised-induced bronchoconstriction [73]. In professional football players, a small to moderate neuroprotective effect of 2 g DHA per day over the course of an American football season was reported [66].

## 4. Discussion

The evidence presented in the studies reviewed here show that EPA/DHA may have the potential to influence not only the metabolic response of muscle to nutrition, but also the physiological functional response to exercise and post-exercise conditions. However, these physiological and metabolic adaptations do not always translate into improved performance.

Based on the review of the literature presented here, there seem to be bigger gains for amateurs. It is possible that there is a genuine difference, that amateurs have more to gain from EPA/DHA supplementation, but it could be due to the higher metabolic flux in athletes meaning they require more EPA/DHA to see the benefits (either in terms of dosage or supplementation duration). Alternatively, it could be the case that studies tend to be longer in the amateur groups (from experience, amateurs are more willing to keep training constantly for longer than athletes), so the positive performance gains are more likely to develop in the longer studies more often seen in amateurs. It should also be acknowledged that the potential for performance gains is narrower in athletes due to the law of diminishing returns.

### 4.1. Increased Performance

It is known that the response of skeletal muscle to exercise can be influenced by the nutritional status of the muscle [74]. This effect is not confined to macronutrients, but EPA/DHA can also potentially influence the exercise and nutritional response of skeletal muscle [75], this in turn can partly explain the observed decrease in soreness. Although the potential for EPA/DHA supplementation to improve muscle mass or function, is supported by mechanistic explanations including structural changes of the muscle cell membranes [76,77] this review found no consistent effect on strength in amateurs. Muscle protein synthesis, a fundamental process in muscle growth, was unchanged after 3.5 g EPA and 0.9 g DHA over 8 weeks, although muscle biopsies revealed that kinase signaling in response to resistance training was altered [31]. However, it has been shown that incorporation of EPA/DHA in muscle cells stimulates foal adhesion kinase, which regulates MPS [78], and may actually have a beneficial effect on muscle protein synthesis. This was shown in amateur females and males in response to anabolic stimuli, in two 8-week intervention studies with 1.86 g EPA and 1.5 g DHA [36,37]. Interestingly, selective improvement in muscle torque and muscle quality after, but not during, exercise was reported in females only [28]. EPA and DHA seemed to optimize the effects of resistance training in amateur elderly females, including dynamic and explosive strength, however these effects did not result in an overall improvement of isometric strength performance [25]. These data corroborate other reports that possibly older adult populations may benefit from EPA/DHA supplementation in the context of preserving muscle mass in an older population [14].

Peroxisome proliferator-activated receptor-gamma coactivator (PGC-1a) is a key regulator of mitochondrial biogenesis. In obese participants, EPA has been shown to stimulate mitochondrial biogenesis [79], which could result in improved endurance regulated via the PGC-1a pathway. It can be hypothesized that lower heart rates and improved O_2_ uptake may lead to better O_2_ delivery to contracting muscles, thereby enhancing endurance performance [80]. Another mechanism for improved endurance could be that EPA/DHA increase the deformability of RBC, which in turn could increase oxygen delivery to the muscles [81]. It has also been shown that exposure of human myotubes to EPA upregulated specific genes that regulate beta-oxidation [82]. Moreover, clinical evidence suggests that EPA/DHA increase fatty acid oxidation via the carnitine palmitoyltransferase-II [38,83]. These mechanisms may well have contributed to the increased fat oxidation during rest by 19% and during exercise by 27% following supplementation of 3 g/day EPA/DHA over 12 weeks in female adults [84].

### 4.2. Enhanced Recovery

The concept of muscle damage following intense eccentric exercise is accepted [85]. The acute exercise recovery period is defined as the initial 96 h following exercise [86]. EPA and DHA have been described to increase the structural integrity of muscle cell membranes [77], which in turn may explain the protective effect of EPA/DHA. This has recently been demonstrated in soccer players where 1.1 g/day EPA/DHA combined with 30 g/day whey protein resulted in reduced levels of muscle soreness along with a reduction of plasma CK concentration [54]. Furthermore, exercise-induced muscle damage causes responses that include DOMS and muscle fatigue. It also leads to increased circulating neutrophils and interleukin-1 peaking within 24 h after the exercise, with skeletal muscle levels remaining elevated for 48 h and longer [87]. Inflammation is a key process in muscular repair and regeneration, the potential of EPA/DHA to accelerate the recovery process via immune modulation come into play. EPA/DHA influence immune modulation via increasing interleukin 2 (IL2) [60,62], where an acute dose of fish oil improved markers of inflammation after eccentric exercise in amateur males [64]. The authors found that in 45 amateur males an acute dose of 1.8 g fish oil before a single eccentric exercise bout lead to a smaller exercise-induced elevation in tumor necrosis factor-α (TNF-α) and prostaglandin (PG)E_2_ immediately, 24 h, and 48 h after exercise, as well as significantly lower elevation in the concentrations of interleukin 6 (IL-6), CK, and myoglobin (Mb) at 24 and 48 h after exercise.

Furthermore, in theory EPA/DHA may contribute to insulin-sensitizing effects because EPA and DHA are natural ligands for peroxisome proliferator-activated receptor γ (PPARγ); following activation of PPARγ, nuclear factor kappa B (NF-κB) activity is suppressed, reducing the release of pro-inflammatory cytokines [88]. At a cellular level, fatty acids have an important function in regulating the activity of certain enzymes and by acting as signaling molecules [3]. It has been shown that 1.3 g fish oil consumption over 6 weeks has the potential to ameliorate the exercise-induced decrease in superoxide dismutase activity in sedentary control participants [59]. In the same study, fish oil tended to increase the catalase activity after 1 h of recovery. Together, these findings suggest that EPA/DHA may activate the superoxide dismutase and catalase pathways. Oxidative stress is usually defined by an increased formation of prooxidants and decrease of antioxidants. This disturbance can lead to oxidative damage to cellular components such as lipids, protein and DNA. However, oxidative stress and inflammation are interdependent. Inflammation can develop following oxidative stress, on the other hand inflammation can induce oxidative stress which further enhances inflammation [89]. Exercise mode, intensity, and duration, as well as the subject population tested, can impact the extent of oxidative stress. Furthermore, the use of antioxidant supplements such as EPA/DHA can impact the outcomes. EPA and DHA have been shown to improve muscle function in older adults [26,27]. A recent paper supports the view that EPA/DHA could bring benefits by attenuating the generation of oxidative stress [90]. In this review, preliminary evidence is provided that EPA/DHA may be beneficial in counteracting exercise-induced inflammation. However, current data are inconclusive as to whether EPA/DHA supplementation at the reported dosages is effective in attenuating the immune-modulatory response to exercise and ultimately improve recovery.

### 4.3. Reduced Risk of Injury/Illness

Optimal sports performance requires optimal health. EIB is a prominent asthma phenotype affecting an estimated 90% of asthma patients and up to 50% of elite athletes [91]. Reduced inflammation ameliorates the severity of asthma and exerts a bronchodilatory effect. The anti-inflammatory effects of EPA/DHA may be linked to a change in cell membrane composition and lipid mediators such as resolvins [92]. Alternatively, the effect may also be mediated by the decreased production of bronchoconstrictive leukotrienes [93].

Certain sports like soccer or rugby can lead to traumatic brain injuries (TBI). As outlined by others, the number of sport-related concussions are increasing globally [94]. Although DHA and EPA have shown promising in vitro and animal evidence of neuronal repair capacities in TBI [95], there has been only one large, controlled intervention study conducted in American football players. The underlying mechanisms for this observation have not been completely elucidated but it is suggested that saturation of brain cells with DHA in particular may facilitate healing after brain trauma, thereby counteracting negative long-term results [96]. Other mechanisms by which specifically DHA could convey neuroprotection include preservation of myelin, alleviation of glutamate cytotoxicity, suppression of mitochondrial dysfunction and down-regulation of alpha-amino-3-hydroxy-5- methyl-4-isoxazolepropionic acid receptor sub- units. Details are discussed elsewhere [94].

This review provides a new angle on the evidence linking EPA/DHA with performance and recovery-related outcomes by analyzing basics in study designs in the context of the training status. The limitations of this analysis include its exploratory nature. The background diet is hardly considered in the studies that were analyzed; therefore, a fundamental confounder cannot be included in this analysis. Other aspects like the sex of study participants were not considered, due to the limited number of studies. Also, the questions of responders vs. non-responders remains unanswered, and all of these aspects need to be addressed in future clinical trials. It cannot be highlighted enough to pay utmost attention to the actual dose and duration when designing clinical trials. Dichotomization is often used in statistical applications to identify thresholds of continuous variables. A recent simulation study suggests that common methods of dichotomization theoretically discover the true threshold [97] albeit with higher numbers of subjects than we had in our review. For the purpose of this review we did not apply subsequent statistical tests but confined ourselves to a descriptive use of dichotomization.

## 5. Conclusions

This review identified evidence to support a role of EPA/DHA in improved performance such as enhanced endurance, markers of functional response to exercise, enhanced recovery or neuroprotection. The majority of evidence stems from studies in amateurs rather than athletes, although most recommendations for EPA/DHA supplementation for improved performance are made for athletes. In practical terms, athletes, and likely more so, amateurs may benefit from the consumption/supplementation of EPA/DHA. The extent to which the different metabolic state, i.e., training status influences the response to the supplementations warrants further research. In general terms there seems to be an effect of supplementation duration, with favorable outcomes appearing more consistently after approximately 6–8 weeks. The same is true for EPA/DHA dosage, with better responses from doses above approximately 1.5–2.0 g/day. Finally, it appears the beneficial outcomes are more consistently seen in amateurs, so broadly speaking the amateurs might require lower doses for a shorter period to experience gains.

It remains to be investigated why the changes in markers do not always lead to measurable improvements in clinical outcomes of performance, recovery and the reduced risk of illness/injury. In a given, well-characterized population the quantity of EPA/DHA and the duration of the supplementation play crucial roles and need to be well defined in order to clearly identify the effects of EPA/DHA on performance. Also, certain questions remain to be investigated such as sex, responders vs. non-responders, or if the habitual intake of EPA/DHA play a role in the efficacy of EPA/DHA for sports nutrition. This exploratory analysis may, therefore, serve as guidance for the basic design of clinical trials that investigate effects of EPA and DHA and avoid pitfalls of study durations that are too short, optimal dose and most importantly the appropriate study population, as training status seems to be a substantial aspect in determining the effects of EPA/DHA supplementation in sports nutrition.

Given that EPA/DHA are considered safe up to 5 g per day there seems little harm in recommending EPA/DHA, even when further larger studies with optimal design are needed to confirm these initial results.

## Figures and Tables

**Table 1:** Effects of EPA/DHA supplementation with a focus on performance-related outcomes in athletes and amateurs.

Population (n) Sex Age ± SD (Years) *	Duration (Weeks)	Dose EPA/DHA (g/d)	Exercise Intervention/Test	Effects of EPA/DHA Compared to Control	Reference
Athletes					
Cyclists (16) M 23.2 ± 1.2 and 27.1 ± 2.7	8 w	0.8 g EPA 2.4 g DHA	Oxygen peak consumption (VO_2_ peak) Sustained submaximal exercise tests at 55% of peak workload	Heart rate (HR), including peak heart rate during incremental workloads to exhaustion, reduced Steady-state submaximal exercise HR, reduced Whole body O_2_ consumption, reduced HR pressure product, reduced VO_2_ peak, no difference Time to voluntary fatigue, no difference	[16]
Cyclists (23) M and F 24.1 ± 2.4 and 26.9 ± 2.8	6 w	2.0 g EPA 0.4 g DHA	10-km time trials	10-km time trial performance, no difference Exercise-induced increases in plasma cytokines, myeloperoxidase, blood total leukocytes, serum C-reactive protein (CRP), and creatine kinase (CK); or the decrease in the salivary Immune (Ig)A:protein ratio, no difference	[17]
Rugby players (20) M 22 ± 0.7	5 w	1.1 g EPA 1.1 g DHA	Pre-season training Muscle soreness countermovement jump (CMJ) performance psychological well-being	Muscle soreness down Explosive power up Fatigue down	[18]
Football players (25) M 21.7 ± 1.0 and 23.2 ± 1.1	5 w	1.9 g EPA + DHA	Endurance performance, Recovery Resting blood pressure (BP) Fasting serum triglycerides (TG) HR during treadmill running at 10 km/h	TG, decreased Diastolic blood pressure, decreased HR during submaximal exercise, decreased Time to exhaustion and recovery, no difference	[19]
Soccer players (26) M and F 24.5 ± 5.0	4 w	4.9 g EPA 1.4 g DHA	During training	Strength, unchanged Power, unchanged Speed, unchanged Anaerobic endurance capacity, increased	[20]
Cyclists (13) M 23.1 ± 5.4	3 w	0.66 g EPA 0.44 g DHA	Nitric oxide (NO) Asymmetric dimethyloarginine (ADMA) Maximal oxygen uptake (VO_2 max_) Flow-mediated dilatation (FMD) Pulse wave velocity	Baseline NO, increased NO concentration (ΔNO), increased Positive correlation between baseline post-intervention NO concentration and VO_2 max_ (r = 0.72; p < 0.01) Positive correlation between ΔNO and ΔVO_2max_ Association between higher FMD and higher ΔVO_2max_, improved	[21]
Cyclists (24) M 19–42	3 w	1.06 g EPA 0.75 g DHA	Endurance test (bicycle time trial of approx. 1 h) Red blood cells (RBC) characteristics and lipid peroxidation	RBC characteristics, no difference Exercise performance, no difference Rate of LDL oxidation, decreased The amount of dienes, no difference Effects of exercise, no difference	[22]
Cyclists (26) M 24 ± 7 and 23 ± 5	8 w	0.56 g DHA 0.14 g EPA	O_2_ consumption and fatigue Isometric quadriceps strength	Power output of maximal 6 s cycle sprinting, unchanged Power output during 5 min time trail (TT), unchanged Maximal voluntary contraction, unchanged Relative O_2_ consumption during the cycling time trial, reduced	[23]
High-intensity intermittent training athletes (27) M 26 ± 4	Acute	Group 1: 0.75 g EPA, 0.05 g DHA per 10 kg body weight Group 2: 0.15 g EPA, 0.1 g DHA per 10 kg body weight	Recovery strategy following 100 plyometric drop jumps	Squat jump performance, increased in group1 CMJ performance, unchanged Functional and perceptual indices, unchanged Perceived soreness, unchanged	[24]
Amateurs					
Healthy (63) F 67.5 ± 0.4	24 w	n-6/n-3 polyunsaturated fatty acids (PUFA) ratio below 2	Resistance training	Isometric strength performance, no difference Dynamic peak power and time to reach peak power (i.e., shorter time) during knee extension, increased Peak force and rate of force development during squat jump, increased	[25]
Healthy (60) M and F 60–85	24 w	1.86 g EPA 1.5 g DHA	Age-associated loss of muscle mass and function Thigh muscle volume, handgrip strength, one-repetition maximum (1-RM) lower- and upper-body strength, and average power during isokinetic leg exercises	Thigh muscle volume, increased Handgrip strength, increased 1-RM muscle strength, increased Average isokinetic power, increased	[26]
Healthy (45) F 64 ± 1.4	21 w 3 d	0.4 g EPA 0.3 g DHA	Muscle strength and functional capacity	The peak torque and rate of torque development for all muscles, increased The activation level and electromechanical delay of the muscles, improved Chair-rising performance, improved	[27]
Healthy (50) M and F 70.6 ± 4.5	18 w	2.1 g EPA 0.6 g DHA	Resistance exercise Training-induced increases in muscle mass and function	Maximal isometric torque increased after exercise training in females, but not in men. Maximal isokinetic torque at 30, 90, and 240° s−1, 4-m walk time, chair-rise time, muscle anatomic cross-sectional area, and muscle fat, no difference Muscle quality in females after exercise training increased no differences in males. No differences in glucose, insulin, or inflammatory markers	[28]
Healthy with decreased muscle mass (53) M and F 74.6 ± 8.0	12 w	0.66 g EPA 0.44 g DHA	Muscle strength Physical performance	Muscle strength, no difference Physical performance, no difference	[29]
Sedentary, overweight (65) M and F 25–65	12 w	1.56 g DHA 0.36 g EPA	Moderate physical activity (3 d/week for 45 min), at 75 % of age-predicted maximal HR. Resting HR and the HR in response to submaximal exercise HR variability	HR variability, improved HR at rest, reduced HR during submaximal exercise, reduced	[30]
Healthy (19) M 24 ± 0 and 21 ± 0 years	8 w	3.5 g EPA, 0.9 g DHA	Resistance exercise Skeletal muscle biopsies were obtained before and after supplementation for assessment of muscle lipid composition and protein kinase activities.	Muscle protein synthesis, no difference Protein kinase B (PKB) activity at rest, reduced PKB and AMP-activated protein kinase a2 (AMPKα2) activity, decreased	[31]
Healthy (20) M 23 ± 1	8 w	0.91 g EPA0.4 g DHA	Maximal O_2_ uptake and oxygen uptake during submaximal exercise	Negative linear correlation in change between erythrocyte EPA and whole O_2_ uptake during submaximal exercise pre- and post-supplementation	[32]
Healthy (21) M 21.0 ± 0.8	8 w	0.6 g EPA 0.26 g DHA	Eccentric strength exercise Motor nerve function Muscle damage Changes in maximal voluntary isometric contraction torque Range of motion Upper arm circumference, Delayed onset muscle soreness (DOMS).	M-wave latency, Maximal voluntary contraction (MVC) torque, higher Range of motion (ROM), greater DOMS, reduced	[33]
Healthy (24) M 19.5 ± 0.8	8 w	0.6 g EPA 0.26 g DHA	Eccentric contraction-induced muscle damage Changes in the MVC torque ROM Upper arm circumference, muscle soreness CK, myoglobin, interleukin-6 (IL-6), and tumor necrosis factor a (TNF-α) levels	MVC 2–5 days after exercise, increased ROM at 1–5 days after exercise, increased Muscle soreness 3 days after exercise, reduced Increase in serum IL-6 levels, reduced	[34]
Healthy (26) M 18–40	8 w	0.56 g DHA 0.14 g EPA	HR, HR variability and HR recovery during rest, intense exercise and recovery	The mean HR during supine resting conditions, no difference HR variability at rest, decreasing trend Peak HR, no difference HR during submaximal exercise, decreased Supine HR recovery (half-time) after cycling, faster	[35]
Healthy (16) M and F 71 ± 2	8 w	1.9 g EPA 1.5 g DHA	Hyperinsulinemic-hyperaminoacidemic clamp	Muscle protein synthesis in older people, increased	[36]
Healthy (9) M and F 39.7 ± 1.7	8 w	1.9 g EPA 1.5 g DHA	Hyperinsulinemic-hyperaminoacidemic clamp	Muscle protein synthesis in young people, increased	[37]
Overweight (50) F 20–45	8 w	0,6 g EPA 0.3 g DHA	Aerobic exercise	VO_2max_, increased	[38]
Healthy (68) M and F 18.6 ± 1.2 and 18.9 ± 1.1	4 w 2 d	FO 2.7 g	Eccentric exercise Omega-3 Index CRP and CK Lactate DOMS, extension and torque Quality of life	Pain following eccentric exercise, reduced Extension or strength, no difference Blood lactate, lower Emotional stability, improved CRP levels at 24 h, reduced	[39]
Healthy (32) M 22.0 ± 2	3 w 5 d	0.06 g EPA 0.04 g DHA	Muscle damaging exercise (downhill running)	Strength loss (MVC), reduced skeletal troponin (sTnI) and TNF-α at 2, 24, 48, 72 and 96 h., Mb at 24, 48, 72, 96 h., reduced CK-MM at all-time, reduced DOMS at 72 and 96 h, reduced Protective effect against joint ROM loss at 96 h Pain, reduced potentiated twitch force (∆Qtw,pot), reduced	[40]
Healthy (30) M 25 ± 4.6	3 w	0.38 g EPA 0.51 g DHA	17 h training/week Maximal voluntary isometric contractions Wingate test 250 kJ time trial	Vastus lateralis, increased Maximal voluntary isometric contractions, no difference Wingate percent power drop, reduced Time trial, no difference	[41]
Healthy (30) M 24.1 ± 3.6 and 24.4 ± 2.6	2 w	0.38 g EPA 0.51 g DHA	Sprint interval training with pre- and post-training TT Resting twitches, quadriceps MVC force, and potentiated twitch force	Maximal voluntary, no difference (∆Qtw,pot, no difference	[42]
Healthy (8) M 24 ± 1	2 w	1.1 g EPA, 0.7 g DHA	VO_2max_, 30 min cycling	Substrate oxidation, no change Energy expenditure, no change Energy efficiency, no change	[43]
Healthy (17) F 22.5 ± 1.8 and 24.7 ± 3.6	1 w	6 g FO (5:1 EPA:DHA)	Post resistance exercise muscle soreness Soreness during functional movements and limb circumferences	Resistance exercise-induced static and functional soreness responses, reduced Static and functional muscle soreness, no difference Upper arm and thigh circumferences, no difference	[44]
Healthy (22) M 23.0 ± 3.6	Acute	50 g high-fat meal (4.7 g EPA) 50 g high-fat meal (4.7 g DHA)	Exercise stress testing Cardiac output, Blood pressure and systemic vascular resistance (SVR)	SVR was lower at 5 h and during exercise following the DHA but not EPA meal Resting cardiac output, no difference 8-iso-PGF2α, no difference Cardiac output during exercise, no difference	[45]

The order of articles is first on athletes then amateurs. Next, they are ordered by supplement duration. M = male, F = female, w = week, d = day, g = gram (numbers are rounded from three to two decimals). * were provided, first for the intervention group, secondly for the placebo group.

**Table 2:** Effects of EPA/DHA supplementation with a focus on recovery-related outcomes in athletes and amateurs.

Population (n) Sex Age ± SD (Years) *	Duration (Weeks)	Dose EPA/DHA (g/d)	Exercise Intervention/Test	Findings	Reference
Athletes					
Football players (15) M 18.9 ± 0.5	8 w	1.14 g DHA	Mitochondria dynamics and antioxidant status in peripheral blood mononuclear cells (PBMC)	PBMCs, Mn-superoxide dismutase protein levels, and their capability to produce reactive oxygen species, no difference Proteins related to mitochondrial dynamics, increased The content in mitofusins (Mtf)-1 and Mtf-2, optic atrophy protein-1 (Opa-1), and mitochondrial transcription factor A (Tfam), increased Cytochrome c oxidase (COX-IV) activity and uncoupling proteins (uncoupling protein) UCP-2 and UCP-3 protein levels, increased	[47]
Football players (15) M 20.4 ± 0.5 and 19.3 ± 0.4	8 w	1.14 g DHA	Pro-oxidant and antioxidant status of peripheral blood mononuclear cells (PBMC)s during training and acute exercise	UCP3 levels after training, increased Superoxide dismutase protein levels after acute exercise, increased Production of reactive oxygen species (ROS) after acute exercise, reduced.	[48]
Football players (15) M 19.7 ± 0.4	8 w	1.16 g DHA	Eicosanoids levels and PBMCs eicosanoids production	Training: Cyclooxygenase 2 (COX-2) protein levels, no difference COX-1 protein levels, increased Acute exercise: COX-2 levels, increased Lipopolysaccharide (LPS)-stimulated PBMCs prostaglandin E (PGE)1 and PGE2 production, decreased Expression of NFκβ, COX-2, 15-LOX2, 5-LOX, or IL-1β genes in PBMCs, no difference	[49]
Football players (15) M 20.4 ± 0.5 and 19.3 ± 0.4	8 w	1.16 g DHA	Cytokine production, by LPS-stimulated PBMCs after exercise	Exercise-induced increase in IL6, IL8, vascular endothelial growth factor , INFγ, TNFα, IL1α, IL1β, MCP1, decreased EGF production rates by LPS-stimulated PBMCs, reduced	[50]
Football players (15) M 19.7 ± 0.4	8 w	1.14 g DHA	Plasma oxidative balance and anti-inflammatory markers after training and acute exercise	Biomarkers for oxidative balance in plasma, no difference During training, plasma protein markers of oxidative damage, haemolysis degree, antioxidant enzyme activities, increased Lipid oxidative damage, no difference PGE2 in plasma after acute exercise, increased	[51]
Football players (15) M 20.4 ± 0.5 and 19.3 ± 0.4	8 w	1.14 g DHA	After training and acute exercise Oxidative balance Oxidative damage markers Activity and protein level of antioxidant enzymes	Enzyme activities in erythrocytes, increased Catalytic activity of superoxide dismutase, increased Peroxidative damage induced by training or exercise, reduced	[52]
Judoists (20) M 22.8 ± 1.4 and 22.3 ± 1.4	6 w	0.6 g EPA 0.4 g DHA	Oxidative stress at rest and after training	Triglycerides, reduced Resting MDA concentrations, increased NO and oxidative stress, i.e., malondialdehyde (MDA), maximum rate of oxidation (Rmax), conjugated dienes (CD)max, and NO), increased Retinol and α-tocopherol, no difference	[53]
Football players (30) M 23 ± 1 year	6 w	0.55 g DHA 0.55 g EPA	Eccentric exercise Physiological markers of recovery measured over three days following eccentric exercise	Muscle soreness, reduced compared to protein Blood concentrations of CK, reduced compared to CHO Muscle function, no difference CRP, no difference	[54]
Taekwondo athletes (10) M 45.6 ± 1.6 and 22.8 ± 3.8	5 w	0.82 g DHA + 0.33 g α-tocopherol	Maximal exercise test	Oxidative and nitrative damage, no change Antioxidant and mitochondrial gene expression, no change	[55]
Taekwondo athletes (18) M 45.6 ± 1.6 and 22.8 ± 3.8	5 w	0.82 g DHA + 0.33 g α-tocopherol	Acute exercise test	Pro-inflammatory gene expression in young increased	[56]
Paddlers M (18) 23.1 ± 1 and 23.6 ± 1.9	4 w	1.2 g DHA [56] 2.4 g EPA	During intense exercise	Production of tumor necrosis factor (TNF)-α, decreased Interleukin (IL)-1β, decreased Production of IL-6, increased Production of interferon (IFN)-γ, decreased Production of IL-10, increased	[57]
Amateurs					
Healthy (27) M 33.4 ± 4.2	8 w	1.8 g FO	Eccentric exercise Knee ROM, perceived pain, and thigh circumference of the right leg	Pain and ROM before, immediately, and 24 h after the exercise, no difference Perceived pain and ROM at 48 h post-exercise, improved	[58]
Healthy (24) M 19.5 ± 0.8	8 w	0.6 g EPA 0.26 g DHA	Eccentric contraction-induced muscle damagemuscle soreness	3 days after exercise, muscle soreness of the brachialis, reduced MVC, increased	[34]
Healthy (24) M 21.0 ± 0.9 and 20.7 ± 1.1	6 w	1.3 g FO	1 h of exercise with a constant work load corresponding to 60% of their individual VO_2max_) followed by a maximal rate Blood antioxidant status and lipid profile	Resting concentration of triglycerides, decreased Superoxide dismutase activity, improved Catalase activity in response to exercise after 1 h of recovery, increased	[59]
Healthy (16) M 24 ± 3.8	6 w	1.3 g EPA 0.3 g DHA	Single bout of exercise, maximal exercise test and a 1-h bout of endurance exercise at 70% VO_2_ peak Plasma IL-6, EPA, DHA and cortisol; PBMC IL-2, IL-4 and interferone (IFN)-γ production; neutrophil phagocytosis/oxidative burst; and natural killer (NK) cell cytotoxic activity	At 3 h post-exercise PBMC, IL-2 and NK cell activity increased PBMC, IL-4 and IFN-γ productions, plasma IL-6 and cortisol concentrations, as well as neutrophil activity, no difference	[60]
Healthy (20) M 23 ± 2.3	6 w	1.3 g EPA 0.3 g DHA	Exercise-induced markers of oxidative stress and muscle damage Eccentric strength exercise	CK, protein carbonyls, endogenous DNA damage, muscle soreness or MVC, unchanged Plasma thiobarbituric acid reactive substances, decreased H_2_O_2_ stimulated DNA damage immediately post-exercise, decreased	[61]
Healthy (37) M and F 25.8 ± 5.3	6 w	0.24 g EPA 0.12 g DHA	Maximal incremental exercise test and cycling TT. Post-exercise immune function and performance Plasma IL-6 and thiobarbituric acid reactive substances (TBARS) concentrations and, erythrocyte fatty acid composition NK cell cytotoxic activity and PBMC IL-2, IL-4, IL-10, IL-17 IFNγ production	PBMC IL-2 and NK cell cytotoxic activity 3 h post-exercise, increased Plasma IL-6 and TBARS, PBMC IL-4, IL-10, IL-17 and IFNγ production, along with performance and physiological measures during exercise, no difference	[62]
Healthy (32) M 22.0 ± 2	3 w 5 d	0.06 g EPA 0.04 g DHA	Muscle damaging exercise (downhill running)	sTnI and TNF-α at 2, 24, 48, 72 and 96 h., Mb at 24, 48, 72, 96 h., reduced CK-MM at all-time, reduced DOMS at 72 and 96 h, reduced MVC, reduced Protective effect against joint ROM loss at 96 h Pain, reduced ∆Qtw,pot, reduced	[40]
Healthy (27) F 33.3 ± 2.4 and 31.9 ± 3.1	1 w 2 d	3 g DHA	Eccentric strength exercise	Increase in soreness was 23% less Number of participants who were able to achieve full active elbow extension 48 h after eccentric exercise was greater in the DHA group No differences for passive elbow extension or arm swelling	[63]
Healthy (11) M and F 18–60	1 w	2 g EPA 1 g DHA	Eccentric strength exercise Inflammation Soreness ratings Arm circumference and volume Temperature	Soreness, decreased Arm circumference, no difference Arm volume, no difference Skin temperature, no difference	[46]
Healthy (17) F 22.5 ± 1.8 and 24.7 ± 3.6	1 w	6 g FO (5:1 EPA:DHA)	Post resistance exercise muscle soreness Soreness during functional movements and limb circumferences	Muscle soreness, no difference	[44]
Healthy (45) M 29.3 ± 6.2 and 31.1 ± 4.9	Acute	1.8 g FO	Plasma levels of PGE2, IL-6, TNF-α, CK, LDH, and myoglobin (Mb) after eccentric exercise	TNF-α and PGE2 immediately, 24, and 48 h after exercise, reduced Elevation concentration of IL-6, CK, and Mb at 24 and 48 h after exercise, reduced Plasma concentration of LDH immediately, 24, and 48 h after the exercise program, reduced	[64]

The order of articles is first on athletes then amateurs. Next they are ordered by supplement duration. M = male, F = female, w = week, d = day, g = gram (numbers are rounded from three to two decimals).*were provided, first for the intervention group, secondly for the placebo group.

**Table 3:** Effects of EPA/DHA supplementation with a focus on injury/illness-related outcomes in athletes and amateurs.

Population (n) Sex Age ± SD (Years) *	Duration (Weeks)	Dose of EPA/DHA (g/d)	Exercise Intervention/Test	Effects of EPA/DHA	Reference
Athletes					
Football players (81) M No age reported	Over the course of a season	2, 4, or 6 g of DHA	Neuroprotection Neurofilament light (NFL)	DHA likely attenuated serum NFL coincident with increases in serum NFL by likely small and moderate magnitude (effect size = 0.4–0.7)	[66]
Endurance athletes with asthma (16) M 30 ± 9 and 25 ± 4	3 w	3.7 g EPA 2.5 g DHA 1.8 g EPA 1.3 g DHA	Eucapnic voluntary hyperpnoea (EVH) challenge	The peak fall in forced expiratory volume (FEV)1 was similarly reduced in both intervention groups compared to placebo (*p* < 0·001). Baseline fraction of exhaled NO was reduced by 24 % (*p* = 0·020) and 31 % (*p* = 0·018) after 6·2 and 3·1 g/d n-3 omega-3, respectively. Peak increases in 9α, 11β PGF2 after EVH were reduced by 65 % (*p* = 0·009) and 56 % (*p* = 0·041) after 6·2 and 3·1 g/d n-3 PUFA, respectively	[67]
Amateurs					
Asthma (20) M and F 22.6 ± 2.1	3 w	0.07 g EPA 0.05 g DHA	EVH challenge	Maximum fall in post-EVH FEV1 significantly reduced (*p* < 0.05) Pre- and post- EVH, EBC ph cyst-LT and 8-isoprostane, and urinary 9a, 11b-PGF2 and CC16 concentrations were significantly reduced (*p* < 0.05)exhaled breath condensate pH (EBC pH) and asthma symptom scores were significantly improved (*p* < 0.05) Rescue medication use significantly reduced (*p* < 0.05)	[68]
Asthma (23) M and F 19–54	3 w	4.0 g EPA 2.0 g DHA	Bronchial hyperresponsiveness to mannitol	No changes in sputum eosinophils No differences in Forced expiratory volume	[69]

The order of articles is first on athletes then amateurs. Next they are ordered by supplement duration. M = male, F = female, w = week, d = day, g = gram (numbers are rounded from three to two decimals). *were provided, first for the intervention group, secondly for the placebo group.
